# CHMP4C Disruption Sensitizes the Human Lung Cancer Cells to Irradiation

**DOI:** 10.3390/ijms17010018

**Published:** 2015-12-24

**Authors:** Kang Li, Jianxiang Liu, Mei Tian, Gang Gao, Xuesong Qi, Yan Pan, Jianlei Ruan, Chunxu Liu, Xu Su

**Affiliations:** Key Laboratory of Radiological Protection and Nuclear Emergency, China CDC, National Institute for Radiological Protection, Chinese Center for Disease Control and Prevention, 2 Xinkang Street, Dewai, Beijing 10088, China; likangtianyi@163.com (K.L.); jxliu@163.com (J.L.); tianmei1203@aliyun.com (M.T.); gaogang@nirp.cn (G.G.); cedar121@sina.com (X.Q.); ruanjl2013@sohu.com (Y.P.); ananpy@163.com (J.R.); liuchunxu0214@126.com (C.L.)

**Keywords:** CHMP4C, Aurora B, human non-small lung cancer cells, radiation

## Abstract

Human lung cancer is highly invasive and the most malignant among human tumors. Adenocarcinoma as a specific type of non-small cell lung cancer occurs with high frequency and is also highly resistant to radiation therapy. Thus, how to avoid radiation resistance and improve radiotherapy effectiveness is a crucial question. In the present study, human lung cancer A549 and H1299 cells were irradiated using γ-rays from a Co60 irradiator. Protein expression was detected by Western blotting. Cell cycle and apoptosis were measured by flow cytometry. Surviving fraction was determined by colony formation assay. γH2AX and 53BP1 foci formation were examined by fluorescence microscopy. In the results, we show that CHMP4C, a subunit of Endosomal sorting complex-III (ESCRT-III), is involved in radiation-induced cellular response. Radiation-induced Aurora B expression enhances CHMP4C phosphorylation in non-small cell lung cancer (NSCLC) cells, maintaining cell cycle check-point and cellular viability as well as resisting apoptosis. CHMP4C depletion enhances cellular sensitivity to radiation, delays S-phase of cell cycle and reduces ionizing radiation (IR)-induced γH2AX foci formation. We found that Aurora B targets CHMP4C and inhibition of Aurora B exhibits similar effects with silencing of CHMP4C in radioresistance. We also confirm that CHMP4C phosphorylation is elevated after IR both in p53-positive and-negative cells, indicating that the close correlation between CHMP4C and Aurora B signaling pathway in mediating radiation resistance is not p53 dependent. Together, our work establishes a new function of CHMP4C in radiation resistance, which will offer a potential strategy for non-small cell lung cancer by disrupting CHMP4C.

## 1. Introduction

Human lung cancer is highly invasive and most malignant among human tumors, which is classified into non-small cell lung cancer and small cell lung cancer. Non-small cell lung cancer accounts for about 85% of lung cancers and the current therapeutic strategy includes surgical resection together with radiation and/or chemo-treatment. Radiation resistance is the severe outcome of lung cancer radiotherapy and therefore poses a critical barrier for the radio-therapeutic effect. Hence, how to avoid the radiation resistance and to improve its treatment efficiency is a major challenge [1,2].

Ionizing radiation initiates DNA damage and arrests cell cycle progression, leading to cellular genomic instability and loss of genetic information. In response to the damage stress, the ataxia telangiectasia mutated kinase (ATM) and ataxia telangiectasia and Rad3-related protein (ATR) are activated. ATM and ATR can also engage in cell division control via activating cyclins and cyclin-dependent kinases (Cdks) [3,4]. A recent study found that in the cell division, there is a very important cytokinetic abscission checkpoint regulated by CHMP4C, which controls abscission time to coordinate mid-body resolution and prevents accumulation of DNA damage. CHMP4C can check the abscission timing through Aurora B-directed phosphorylation [5].

Aurora B kinase is the key kinase of CPC (Chromosomal Passenger Complex) essential for the mitotic processes. Aurora B expression and activity are altered during the cell cycle and peaks at the G2-M transition. Aurora B is elevated in a variety of human tumors and its over-expression is susceptible to tumor formation and poor outcomes of non-small cell lung cancer radiotherapy, therefore indicating that Aurora B might be a drug target for lung cancer [6,7,8]. However, whether Aurora B directly participates in lung cancer development is still unknown.

That ESCRT (Endosomal sorting complex) is required for the CPC (Chromosomal passenger complex)-mediated cytokinetic abscission, during which CPC monitors the right abscission time of two daughter cells. ESCRT has six distinct complexes (containing ESCRT-0, -I, -II, -III, ALIX and Vps4) engaging in multi-vesicular body formation, cytokinesis and HBV (Hepatitis B virus) budding [9,10,11]. ESCRT-III (Endosome sorting complex-III) mediates membrane fission at the end of cytokinesis. A recent study reported that CHMP4C, a subunit of ESCRT-III, retards abscission and inhibits DNA damage accumulation in abscission checkpoint [12,13,14,15]. Meanwhile, CHMP4C enhances autophagy and endosome production, during which p53 transcriptionally regulates CHMP4C via binding the CHMP4C promoter from −512~−450 DNA sequence in response to stress [16,17]. As the common target of Aurora B and p53, CHMP4C has lower expression in normal tissues and high expression in cancers [18]. Overall, despite CHMP4C is involved in abscission checkpoint and autophagy, how it serves its function in DNA damage response as well as in lung cancer formation still remains unclear.

In the study, we first demonstrate that the subunit of ESCRT-III CHMP4C is involved in cellular radiation reactions. Radiation enhances Aurora B expression and CHMP4C phosphorylation in NSCLC cells, collectively directing cell cycle check-point and promoting cell survival. CHMP4C silencing increases cellular sensitivity to radiation, hinders S-phase progression in cell cycle and diminishes ionizing radiation (IR)-triggered γH2AX foci formation. We show that CHMP4C is the major target of Aurora B and has a similar effect with Aurora B upon radiation. We further reveal that the phosphorylation level of CHMP4C is rising both in p53-positive and-negative cells, showing that the close correlation between CHMP4C and Aurora B signaling pathway in mediating radiation resistance is not p53 dependent. Overall, our work discovers a novel action of CHMP4C in radiation resistance, suggesting CHMP4C as a new drug target for non-small cell lung cancer treatments.

## 2. Results

### 2.1. Radiation Induces CHMP4C Expression and Phosphorylation

**Figure 1:**
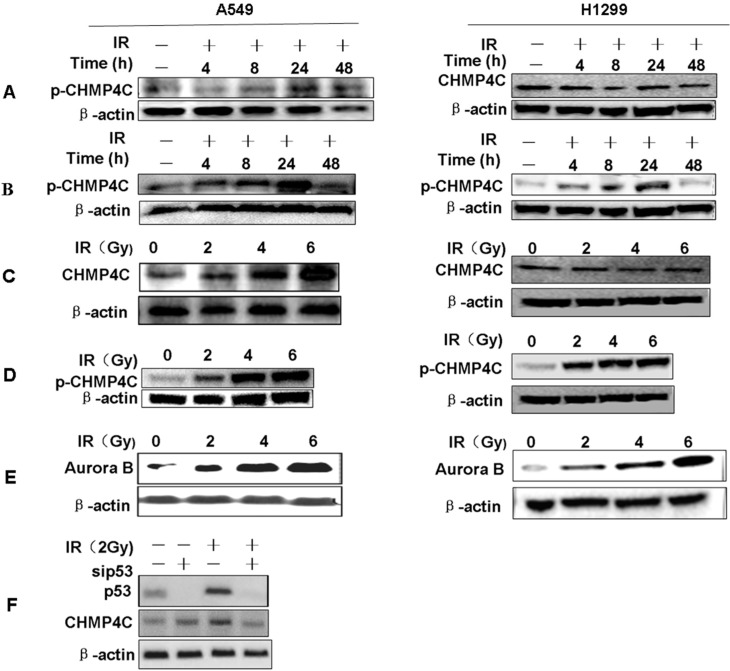
Radiation induces CHMP4C expression and phosphorylation. (**A**,**B**) The A549 or H1299 cells were irradiated with 2 Gy, and harvested at 4, 8, 24 and 48 h. CHMP4C expression or phosphorylation level was tested by Western blot using indicated antibodies; (**C**,**D**) Western blot analysis of CHMP4C expression or phosphorylation level at 24 h after treated with 2, 4, and 6 Gy IR in A549 or H1299 cells; (**E**) Aurora B expression level was tested by Western blot at 24 h after cells exposure to 2, 4, and 6 Gy IR; (**F**) Western blot analysis of expression of CHMP4C and P53 after p53 silencing with or without IR (2 Gy) in A549 cells.

To test if CHMP4C is involved in DNA damage response, cells were γ-irradiated with 2, 4, and 6 Gy and CHMP4C expression as well as phosphorylation was checked. We found that CHMP4C protein increases after irradiation (IR) and peaks at 24 h in A549 but not in H1299 cells (Figure 1A,B). However, the level of CHMP4C phosphorylation and Aurora B expression both increase with IR in both A549 and H1299 (Figure 1C–E). Further, we detected p53 and CHMP4C expression after p53 silencing with or without IR (2 Gy). We found that CHMP4C protein did not increase after irradiation in p53-cleaned A549 cells (Figure 1F). These data maybe indicate increased CHMP4C protein level relies on p53 whereas its phosphorylation is just dependent of Aurora B.

### 2.2. CHMP4C Silencing Delayed S-Phase of the Cell Cycle

**Figure 2:**
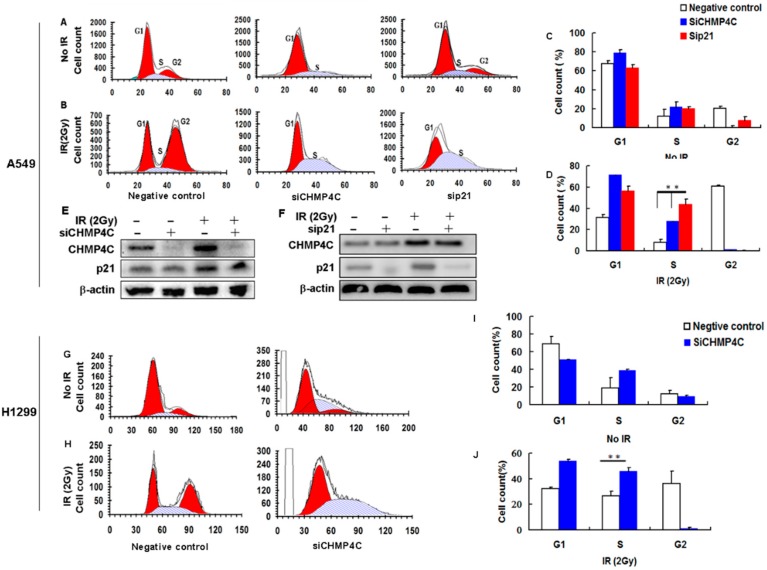
CHMP4C silencing delayed S-phase of the cell cycle. (**A**,**B**) A549 cells were transfected with negative control, siCHMP4C or sip21 for 24 h and/or exposed to 2 Gy IR. After 24 h, the cells were stained with Vibrant Dyecycle green stain and tested on a flow cytometer; (**C**,**D**) Cell cycle analyses of A549 cells. The data are presented as the mean ± S.E. of three independent experiments, ** *p* < 0.01; (**E**,**F**) Western blot analysis of Expression of CHMP4C and P21 after CHMP4C or p21 silencing with or without IR (2 Gy); (**G**,**H**) H1299 cells were transfected with negative control or siCHMP4C for 24 h and/or exposed to 2 Gy IR; After 24 h, the cells were stained with Vibrant Dyecycle green stain and analyzed on a flow cytometer; (**I**,**J**) Cell cycle analyses of H1299 cells. The data are presented as the mean ± S.E. of three independent experiments, ** *p* < 0.01.

Next, we wondered if CHMP4C exerts its regulatory role in cell cycle progression. A549 and H1299 cells were depleted of CHMP4C for 24 h and the S phase delay was checked, indicating that CHMP4C could impact S phase exit (Figure 2A–D,G–J). Further, to identify whether CHMP4C depletion could enhance the radio-sensitivity to A549 and H1299 cells, we analyzed the cell cycle of A549 and H1299 cells cleared of CHMP4C after IR. When A549 and H1299 cells were treated with 2 Gy of radiation, G2/M transition was severely blocked and S-phase was retarded, whereas combined radiation and CHMP4C knockdown only results in S-phase delay. In the meantime, similar results were found in p21-cleaned A549 cells (Figure 2A–D), implying that CHMP4C might overlay or replenish the p21 function in cell cycle checkpoint during DNA damage response in A549 cells.

**Figure 3:**
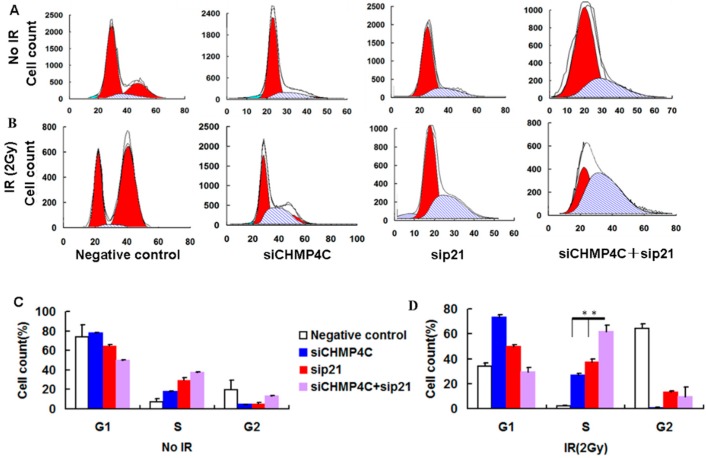
CHMP4C and p21 double silencing exerts an additive effect in S-phase delay of the cell cycle in A549 cells. (**A**,**B**) A549 cells were transfected with negative control, siCHMP4C, sip21, or double siCHMP4C and sip21 for 24 h and/or exposed to 2 Gy IR. After 24 h, the cells were stained with Vibrant Dyecycle green stain and tested on a flow cytometer; (**C**,**D**) Cell cycle analyses of A549 cells. The data are presented as the mean ± S.E. of three independent experiments, ** *p* < 0.05.

In addition, CHMP4C knockdown has no influence on p21 expression (Figure 2E), and p21 depletion cannot effect CHMP4C expression (Figure 2F), which reveals that although CHMP4C and P21 are both p53 target genes, they may be in the different signaling pathway. The double siRNA experiments show that both CHMP4C and p21 depletion exerts an additive effect in the S phase exit (Figure 3), suggesting that CHMP4C might overlay or replenish the p21 function in cell cycle checkpoint during DNA damage response in A549 cells, by participating in different signaling pathways.

### 2.3. CHMP4C and Aurora B Increase Radioresistance

**Figure 4:**
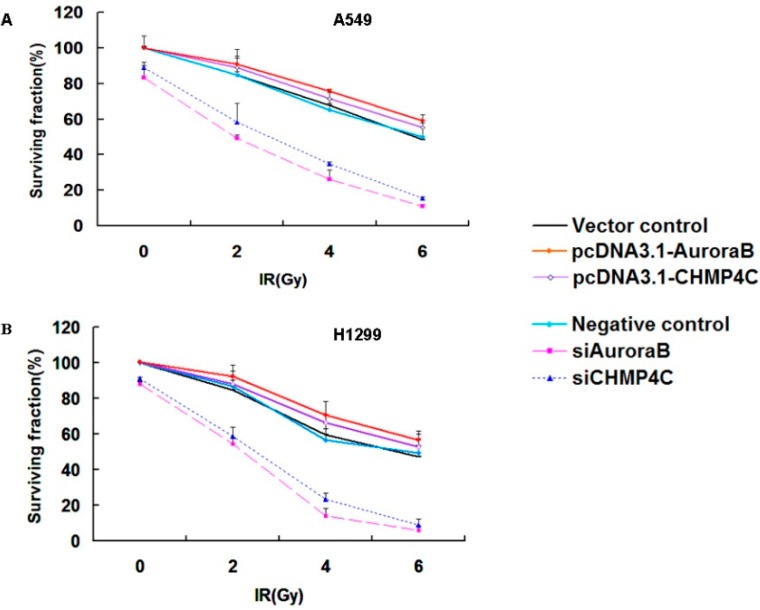
CHMP4C and Aurora B increase radioresistance. (**A**) A549 or (**B**) H1299 cells were transfected with siRNAs or plasmids for 24 h before exposed to 2, 4, and 6 Gy-irradiation. One thousand cells per plate were seeded immediately after IR and incubated for 14 days. Colonies were stained with 1% crystal violet. The number of colonies was counted and surviving fraction was calculated as the mean number of colonies/(cells seeded × plating efficiency). The data are expressed as the mean ± S.E. of three independent experiments.

The above data indicate that CHMP4C inhibition can arrest S-phase of cell cycle. Colony formation assays were next employed to detect if CHMP4C acts in cell radioresistance. These assays showed that CHMP4C knockdown decreases cell survival compared to control with IR. Similarly, the result was also found in Aurora B clearing. Inversely, CHMP4C or Aurora B expression can increase cell growth upon irradiation (Figure 4A,B).

### 2.4. CHMP4C Acts Downstream of Aurora B

**Figure 5:**
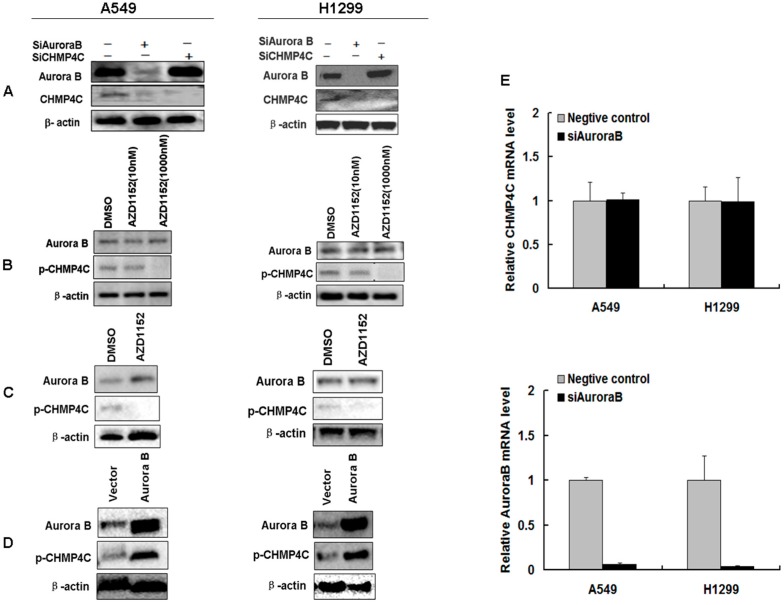
CHMP4C acts downstream of Aurora B. (**A**) Expression of Aurora B and CHMP4C after Aurora B or CHMP4C knockdown in A549 and H1299 cells; (**B**) The phosphorylation level of CHMP4C in untreated and AZD1152 treated (10 and 1000 nM) A549 and H1299 cells; (**C**) The phosphorylation level of CHMP4C in untreated and AZD1152 treated (100 nM) A549 and H1299 cells; (**D**) Western blot analysis of CHMP4C phosphorylation level in A549 and H1299 cells transfected with pCDNA3.1-Aurora B; (**E**) The mRNA expression level of CHMP4C or Aurora B was quantitated with real-time PCR (mean ± S.E., *n* = 3) after the A549 and H1299 cells undergone transfection of negative control or siAurora B.

To verify whether Aurora B regulates CHMP4C, we silenced or overexpressed the Aurora B with siRNA or Aurora B expression plasmids in A549 or H1299. CHMP4C knockdown has no effect on Aurora B protein level, whereas Aurora B silencing inhibits CHMP4C protein level indicating that Aurora B acts upstream of CHMP4C (Figure 5A). Moreover, Aurora B overexpression leads to increased CHMP4C phosphorylation and inhibiting Aurora B kinase activity using phosphorylation inhibitor AZD1152-HQPA reduces CHMP4C phosphorylation (Figure 5B–D). We then found the CHMP4C mRNA level was unchanged following Aurora B downregulation (Figure 5E), further making sure that Aurora B regulates the CHMP4C at the protein level. Collectively, these data suggest that Aurora B modulates CHMP4C expression at the protein level.

### 2.5. CHMP4C Silencing Increases Cell Apoptosis with Irradiation

**Figure 6:**
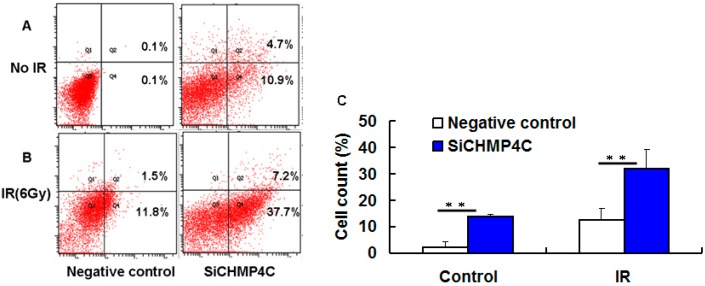
CHMP4C silencing increases cell apoptosis with irradiation. (**A**) CHMP4C-defective cells without IR; (**B**) siRNA transfected cells were exposed to 6 Gy of irradiation. (**C**) Quantification of cell apoptosis assays. The data are presented as the mean ± S.E. of three independent experiments. ** *p* < 0.01

The results of colony formation assay indicate that the surviving fraction of NSCLC cells was significantly attenuated after IR treatment of 4, 6 Gy. We next want to ascertain whether CHMP4C knockdown can enhance IR-induced apoptosis under three conditions in A549 cells: radiation only (6 Gy), CHMP4C knockdown, combined CHMP4C knockdown and IR (6 Gy). We found that combined CHMP4C silencing and irradiation produce more evident apoptosis than single treatment (Figure 6). Hence, these assays reveal that CHMP4C can facilitate cellular proliferation and radiation resistance, further confirming the colony formation assays.

### 2.6. CHMP4C Reduction Suppresses IR-Induced γH2AX and 53BP1foci Formation

**Figure 7:**
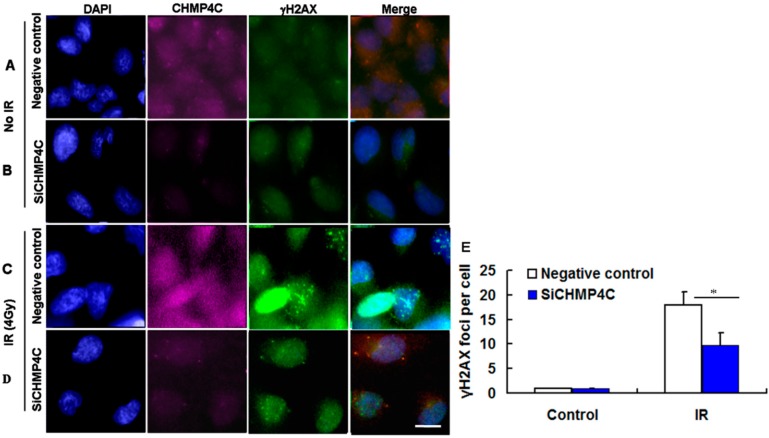
CHMP4C reduction suppresses IR-induced γH2AX foci formation. (**A**,**B**) Negative control and siCHMP4C transfected cells without IR; (**C**,**D**) A549 cells with or without CHMP4C knockdown were exposed to 4 Gy radiation; and (**E**) image of γH2AX foci formation. The number of foci per nucleus was quantitated. The data are presented as the mean ± S.E. of three independent experiments, * *p* < 0.05. Scale bar, 10 µm.

**Figure 8:**
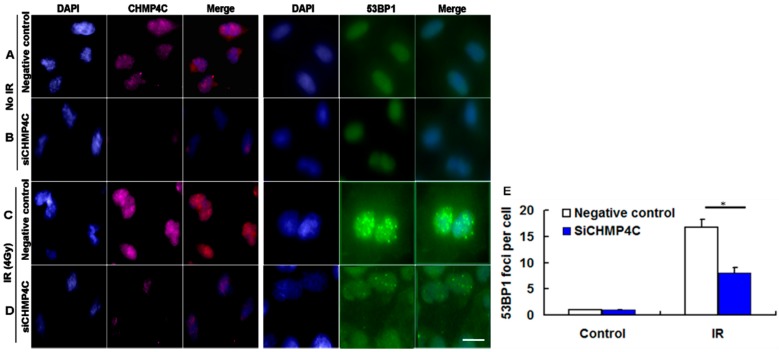
CHMP4C abrogation represses IR-induced 53BP1 foci formation. (**A**,**B**) Negative control or siCHMP4C transfected cells without IR; (**C**,**D**) A549 infected with negative control or siCHMP4C were then treated with 4 Gy radiation; and (**E**) image of 53BP1 foci formation. The number of foci per nucleus was quantitated. The data are presented as the mean ± S.E. of three independent experiments, * *p* < 0.05. Scale bar, 10 µm.

Next, to analyze if CHMP4C is immediately involved in DNA damage signaling, we examined the γH2AX and 53BP1 foci assembly in A549 cells with or without CHMP4C silencing. The IR dose of 4 Gy was selected in this analysis according to the results of colony formation and apoptosis assay. As shown in Figure 6, the indicated dose (4 Gy) of γ-irradiation is sufficient to induce γH2AX and 53BP1foci formation in nuclear (Figure 7C,D and Figure 8C,D). The results demonstrated that during 4 Gy of irradiation, CHMP4C reduction decreased the intensity and number of γH2AX and 53BP1foci compared with the normal CHMP4C, proving that CHMP4C repression indeed represses recruitment of γH2AX, 53BP1 and other related proteins to the DNA lesions (Figure 7 and Figure 8).

## 3. Discussion

**Figure 9:**
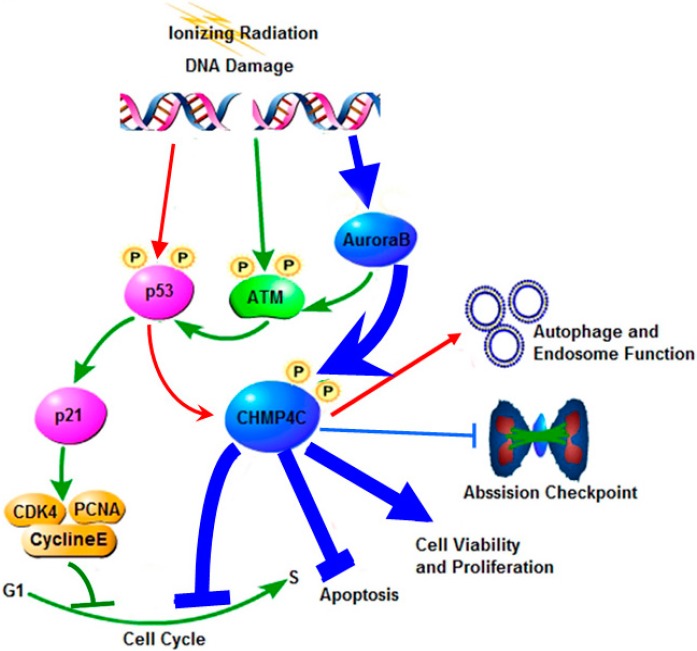
The summary of CHMP4C signaling in response to stress. CHMP4C is phosphorylated by Aurora B to regulate the abscission timing checkpoint and cell cycle progression, promote cell survival and cell viability and resist apoptosis (Blue arrows). The ataxia telangiectasia mutated kinase (ATM) is activated in mitosis in Aurora B-dependent manner (Green arrows). p53 transcriptionally regulates CHMP4C to enhance autophagy and endosome production (Red arrows). p21 is the target gene of p53 and functions in the G1 to S checkpoint (Green arrows).

Current investigations on CHMP4C mainly focus its function on endosome generation. However, whether and how it serves its part in cell cycle, viability and apoptosis under irradiation is not clear. It has been reported that p53 regulates transcription of CHMP4C in the autophagy and endosome production [16]. p53 plays a critical role in regulating DNA-damage-associated cell cycle progression, DNA repair, or apoptosis in the p53/p21 pathway. As the classic target gene of p53, p21 regulates the cyclin-Cdk and PCNA complexes required for the G_1_ to S transition [19,20,21]. We then supposed that they might have similar function in p53-directed cell cycle switch. In support of this hypothesis, our results indicated that CHMP4C knockdown was engaged in cellular S-phase delay, showing the similar regulation with p21 in cell cycle progression in A549. Moreover, double knockdown of CHMP4C and p21 produced an additive effect in S-phase delay of the cell cycle. However, CHMP4C knockdown has no influence on p21 expression and p21 depletion cannot effect CHMP4C expression, which reveals that the novel role of CHMP4C in cell cycle shows an alternative pathway paralleling the p21 signal pathway (Figure 9).

Further, our results also showed that CHMP4C can promote cell survival and cell viability upon IR, indicating that CHMP4C offer the radioresistance to the irradiated cells. We next studied if CHMP4C was connected with DNA repair, and found that CHMP4C inhibition repressed DNA repair by decreasing γH2AX and 53BP1 foci reacting to radiation stress. Hence, the novel functions of CHMP4C in cell cycle control and DNA repair not only enriches the content of the DNA damage pathway but also extends its specific role in cell regulation.

p53 is often mutated in NSCLC and its mutation increases sensitivity to ionizing radiation in tumor cells [22]. CHMP4C transcription is controlled by p53 in endosome function [16]. CHMP4C functions in the Aurora B-dependent abscission checkpoint and inhibits abscission upon phosphorylation by Aurora B, eventually preventing premature resolution of intercellular chromosome bridges and DNA damage accumulation. Our results reveal that increased Aurora B kinase enhances the CHMP4C phosphorylation after IR, which is independent of p53. This is also supported by the facts that the cell lines displayed higher sensitivities to IR after CHMP4C inhibition compared to IR alone, and CHMP4C can increase cell survival both in A549 and H1299 cells.

Several Aurora kinase inhibitors are tested in clinical trials [23]. Studies have shown that inhibition of Aurora B radiosensitizes tumor cells [6,7]. In the study, we observed the reduction of CHMP4C protein level but its mRNA expression remains unchanged following silencing of Aurora B, revealing that Aurora B might regulate the CHMP4C protein stability. Inhibited Aurora kinase B activity by AZD1152 suppresses CHMP4C phosphorylation. However, overexpression of Aurora kinase B upregulates CHMP4C phosphorylation because of the large amount of Aurora B proteins. Furthermore, CHMP4C suppression also radiosensitizes NSCLC cells similar to that during Aurora B inhibition. We demonstrate that Aurora B kinase is essential to phosphorylate CHMP4C and the role of CHMP4C in radioresistance is dependent on Aurora B, which proves that CHMP4C is the major downstream target of Aurora B. Therefore, we identify the close relation between CHMP4C and Aurora B signaling pathway in mediating radiation resistance in NSCLC cells, which is independent on p53.

CHMP4C can check the cytokinetic abscission to prevent premature and accumulated DNA damage as an abscission timer. CHMP4C deficiency generates the defective abscission check, which makes injured cells quickly pass through M to S phase, leading to DNA repair disability and genomic instability. These data suggests that CHMP4C deficiency disorganizes the cell cycle and enhances cell sensitivity to radiation, thus providing a new combined strategy to diagnosis and treatment of the lung cancer.

We propose that ATM is activated by Aurora B, followed by p53-mediated activation of CHMP4C. In turn, CHMP4C promotes cell viability and proliferation. However, there is another major tumor suppressor factor, the alternative reading frame (ARF), which is negatively regulated by ATM in a transcription-independent manner. Contrary to the function of CHMP4C on cell fate, inhibition of ATM enhances ARF levels and stimulates the tumor-suppressive effects of ARF in human oncogene-transformed and cancer cells [24]. These findings provide insights into our study and broaden the aspects of our further research.

## 4. Materials and Methods

### 4.1. Cell Culture and Irradiation

The human NSCLC cell lines A549 (p53 wild type) and H1299 (p53 deficient) (Cell resource center, Peking union medical college, Beijing, China) were cultured in RPMI-1640 containing 10% fetal bovine serum (Invitrogen, Carlsbad, CA, USA), incubated in 37 °C humidified incubator with 5% CO_2_ and treated with different radiation of 2, 4, and 6 Gy using Co_60_ γ-rays with a dose rate of 1 Gy/min in the Irradiation (IR) Center (Beijing Radiation Center, Beijing Academy of Science and Technology, Beijing, China).

### 4.2. siRNAs and Plasmids Transfection

A549 and H1299 cells were grown at 80% confluence and then introduced with siRNAs against CHMP4C (Ambion, Austin, TX, USA), Aurora B (Ambion), p53 and p21 (GenePharma, Beijing, China) as well as control siRNA (Ambion). CHMP4C, Aurora B, p53 and p21 siRNA sequence is respectively: 5′-CCUGCGUCUCUACAACUAU-3′, 5′-CAUGGAUCUGAACAAAAUATT-3′, 5′-CUACUUCCUGAAAACAACG-3′ and 5′-CCUCUGGCAUUAGAAUUAUTT-3′. siRNA tansfections were performed using Lipofectamine RNAi MAX reagent (Invitrogen). Twenty-four hours after transfection, the cells were irradiated and harvested.

pcDNA3.1-CHMP4C or -Aurora B was contrasted and transfected into A549 or H1299 cells using Lipofectamine 3000 reagent (Invitrogen). Twenty-four hours later, cells were collected and performed for subsequent experiments as siRNAs treatment.

### 4.3. Kinase Inhibition Assays

Aurora B kinase inhibitor AZD1152-HQPA was dissolved in dimethyl sulphoxide (DMSO). The A549 and H1299 cells were treated with AZD1152-HQPA at the concentration of 10, 100 and 1000 nM. After 24 h of treatment, CHMP4C phosphorylation was examined by Western blot.

### 4.4. Western Blot

The cells were harvested and lysed in RIPA lysis buffer (Thermo Scientific Pierce, Waltham, MA, USA). The protein was collected at 12,000× *g* for 15 min at 4 °C and measured by BCA protein assay kit (Thermo Scientific Pierce). Equal amounts of protein were separated on 10% sodium dodecyl sulfate (SDS)-polyacrylamide gels and blotted on nitrocellulose membranes for Western blot analysis. The membranes were blocked in 5% nonfat milk and then incubated with the following primary antibodies: CHMP4C (Abcam, Cambridge, UK), phosphorylated (p)—CHMP4C (Abmart, Arlington, MA, USA), Aurora B (Abcam), p53 (Cell Signaling Technology, Boston, MA, USA), p21 (Cell Signaling Technology) and β-actin (Cell Signaling Technology). The CHMP4C antibody is diluted in 1:500, and the rest were used in 1:1000 dilutions. Membranes were washed in tris-buffered saline containing 0.5% tween-20 and then incubated with goat anti-rabbit lgG (Abcam, 1:2000) or goat anti-mouse lgG (Abcam, 1:3000) conjugated to horseradish peroxidase for 1 h at room temperature. The membranes were detected using Chemiluminescence liquid (Thermo Scientific Pierce) according to the manufacturer’s protocol and analyzed by the Image J software (Bio-Rad, Hercules, CA, USA).

### 4.5. Real-Time PCR

Total RNA was extracted using SV total RNA isolation system kit (Promega, Madison, WI, USA) followed by reverse transcription using the GoScript reverse transcription system kit (Promega). The subsequent cDNA products were used as templates to perform the real-time PCR assays. The primers for the amplification of Aurora B or CHMP4C are as follows; Aurora B forward: TTTGAGATTGGGCGTCCTCT and reverse: CGCCCTCCTTCTCTATCTGG; CHMP4C forward: AGAAGCCCTGGAGAACTCAC and reverse: CTTGGGCAGTATCCTGTTGC. The β-actin was used as the internal control using primers forward: TGCCAGAAAACAAGATGAG and reverse: CACCTTCACCGTTCCAGTTT. PCR amplifications were performed in triplicate wells and each experiment was repeated for three times. The relative expression levels of genes were analyzed through the use of the 2^−∆∆*C*t^ method.

### 4.6. Cell Cycle Assay

Cells were harvested and treated with Vibrant Dyecycle green stain (Invitrogen) at 37 °C for 30 min in a dark place. Then, cell cycle was analyzed on a flow cytometer using 488-nm excitation and green emission.

### 4.7. Colony Formation Assay

To analyze colony formation, Single cell was plated in the 6 cm dish at a density of 1000 cells/mL for 10 to 14 days to form spheres. Colonies were fixed with 4% paraformaldehyde for 20 min and then stained with 1% crystal violet for 15 min. Colonies were counted and surviving fraction was calculated as the mean number of colonies/(cells seeded × plating efficiency).

### 4.8. Cell Apoptosis Assay

Cells were harvested and washed twice with cold PBS. Cells were resuspended in 1× binding buffer (BD) at a concentration of 1 × 10^6^ cells/mL and stained with PE AnnexinV and 7-AAD (BD) for 15 min at room temperature in the dark. Samples were analyzed by flow cytometry.

### 4.9. γH2AX and 53BP1 Foci Formation

Cells were seeded on the glass sheets into 6-well plates. Cells were transfected with siRNAs and then exposed to 4 Gy of irradiation. 30 min later, cells were washed twice in PBS, and then incubated with anti-γH2AX (Millipore, FITC conjugate, Billerica, MA, USA), anti-53BP1 (Abcam) or anti-CHMP4C antibody at a dilution of 1:200 overnight at 4 °C. After washing twice in washing buffer (0.05% Tween-20 in PBS), the cells were incubated with goat anti-rabbit lgG (Abcam, Alexa fluor conjugate) at 37 °C for 1 h. Cells were washed twice with washing buffer and once with PBS. The coverslips were then mounted with mounting medium for fluorescence with DAPI (Vectashield). The image of γH2AX and 53BP1 foci was detected using a Leica DMRXA fluorescent microscope (Leica, Wetzlar, Germany). γH2AX and 53BP1 foci were counted in cells with more than five foci.

### 4.10. Statistical Analysis

Data are presented as the mean ± S.E. of three independent experiments, and the statistics were analyzed by students’ *t* test using Microsoft Excel (Microsoft Campus, Redmond, WA, USA). *p*-value <0.05 indicates statistical significance.

## 5. Conclusions

In conclusion, we identify that CHMP4C is a new radiation-inducible protein engaged in cell cycle and cell survival. We first confirm CHMP4C offers lung cancer cells radioresistance, which will provide a potential radio-therapeutic strategy for non-small cell lung cancer by disrupting CHMP4C expression. In future research, the role of CHMP4C combined with radiation *in vivo*, for example the function of CHMP4C in xenografts [24], will be investigated, which would provide *in vivo* evidence for the new action of CHMP4C.
